# Belief of agency changes dynamics in sensorimotor networks

**DOI:** 10.1038/s41598-018-37912-w

**Published:** 2019-02-13

**Authors:** Verena N. Buchholz, Nicole David, Malte Sengelmann, Andreas K. Engel

**Affiliations:** 1 Department of Neurophysiology and Pathophysiology, University Medical Center Hamburg-Eppendorf, Martinistr. 52, 20246 Hamburg, Germany; 2 Department of Psychiatry and Psychotherapy, Friedrich-Alexander University Erlangen-Nürnberg (FAU), Schwabachanlage 6, 91054 Erlangen, Germany

## Abstract

Controlling an event through one’s own action usually induces a sense of agency, a feeling that arises when an expected outcome matches the intention. The neural correlates of this feeling remain controversial however, as experimental manipulation of the action-outcome chain often introduces mismatch or prediction errors that strongly correlate with the sense of agency. Here, we took a different approach and manipulated the causal belief (self-attribution vs. computer-attribution) by external cues during matched visuo-motor tapping conditions. With magneto-encephalography, we studied the sense of agency from a network perspective, investigating in source space the modulation of local population activity and changes in functional connectivity with motor cortex. Our results show that during the belief of agency primary motor cortex (M1) shows stronger functional connectivity (mediated by the beta band) to inferior parietal lobe and right middle temporal gyrus (MTG). Furthermore, the local feed-forward activity (gamma band power) in extrastriate body area and MTG disappears with that belief. After changes in action context, left M1 shows stronger connectivity in the alpha band with right premotor cortex and left insular-temporal cortex a network that might support active inference in social action context. Finally, a better tapping performance in this rhythmic task was related to alpha power modulations in the bilateral cerebellum and bilateral fusiform body-area, with power suppression during a more precise performance. These findings highlight the role of multiple networks supporting the sense of agency by changing their relative contribution for different causal beliefs.

## Introduction

The sense of agency refers to the sense of oneself as the agent, i.e., the initiator, executor and controller, of one’s own actions or movements and the associated effects in the outside world^1^ —a fundamental aspect or self-experience. Studies using neuroimaging techniques have now shed light on the functional architecture of networks underlying the sense of agency, identifying potential hubs such as posterior and inferior parietal lobe (IPL), cerebellum, ventral premotor (vPMC) and supplementary motor cortex (SMA), the insula, as well as posterior middle and superior temporal cortices (MTG, STG) and, extending to occipital, the extrastriate body area (EBA) (e.g., for an overview see the following reviews^2–5^).

The available studies are typically characterized by (i) the manipulation of sensorimotor contingencies, introducing temporal, spatial or conceptual noise into the “intention-action-effect chain”^6^ -for example tapping either synchronously to a rhythmic tone or causing tones by rhythmical tapping with varying delays^7^, by (ii) varying motor efference copies or intention in active vs. passive movement^8,9^ or by (iii) employing more “cognitive” paradigms, such as priming or other extrinsic cues about agency^10–14^. The first two approaches are mainly based on the influential view that the sense of agency relies on basic motor control principles (i.e., the forward model and efference copy) and on the monitoring or comparison of motor signals with anticipated sensory effects^15,16^. The sense of agency is supposed to arise from the contingency of intentions, motor predictions and the sensory feedback or action outcome. An incongruence between those, also conceptualized as a special kind of prediction error^17,18^, might affect the sense of agency and result in an attribution of an action or action effect to an external source or other agent. Due to the nature of these experimental manipulations, however, it remains controversial whether neural correlates identified with these paradigms actually reflect agency-unspecific processes such as mismatch detection, prediction error or even attentional orienting^18–22^ or are causally involved in the sense of agency^23^.

The third type of studies rather manipulated the attribution of an event at a cognitive level, suggesting that the sense of agency cannot be fully explained by motor control mechanisms but should accommodate a more conceptual level beyond a pre-reflective sensorimotor level^24–26^. In line with this, recent approaches assume that multiple cue types are integrated in this process predictively and postdictively^5,8,17,26^. For example, the active inference theory of the sense of agency assumes different hierarchical levels—and, eventually, also time scales and neural networks^27,28^ —at which predictions and comparisons involved in the sense of agency are formed^5^. These processes are then weighted against each other to minimize prediction errors, and a balanced convergence across the levels results in an estimate of the most likely source of sensation^5,17,26^. Thus, the current viewpoint assumes interactions between sensorimotor processes and intentional factors involved in agency estimation. In tasks, where agency has to be inferred by the subject on the basis of sensorimotor contingencies, a bias towards judgement of agency has been observed, with changes in decision criterion and a suppression of error detection for self-attribution^29,30^. Acting on a lower level, sensory attenuation seems to emerge already during action-selection -potentially via efference copies- and influences discrimination performance during action-outcome observation^31–33^.

**Figure 1:**
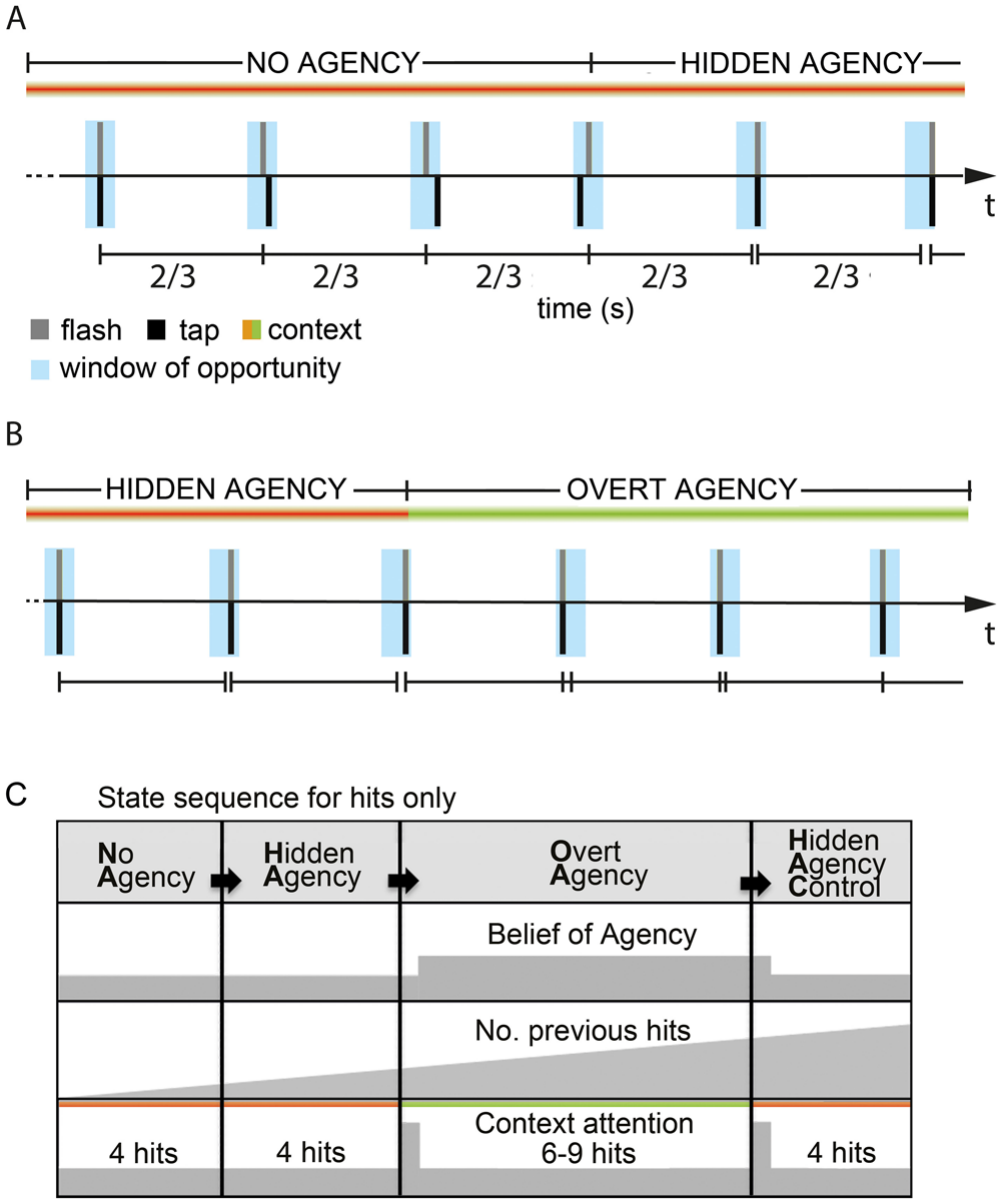
Experimental task. Participants were asked to fixate a rhythmically flashed light stimulus and tap synchronously with this stimulus such that the impact of their right index finger on the armrest coincides with the flash. (**A**) After 4 successive hits inside the window of opportunity (blue, 75 ms duration) in the No-Agency (NA) state, the participant entered the Hidden Agency (HA) state, already triggering the flash (grey) time-locked to the tap (black), seen here in the last two taps. Now the windows of opportunity were adjusted to the previous tap (centered at 2/3 s post-tap). Importantly, there was no change in context light yet, thus the participant was not aware of being agent over the flash. A miss would directly switch the state back to NA from any other state. (**B**) After 4 succesive hits in the HA state, the Overt Agency OA state started and agency was indicated to the participant by a color change in the context light. The flash was still locked to the tap as before. After another 7–9 hits, the HA state was repeated Hidden Agency Control (HAC), by falsely indicating loss of agency for 4 succesive hits (not shown here) and then again with NA. Here the participant initially tapped slightly slower, then faster than 1.5 Hz, but stayed within the window of opportunity. (**C**) Participants passed sequentially through NA, HA, OA, and HAC as long as they managed to tap without misses. Grey indicates hypothesized neural activity for a given process (belief of a agency changing shortly after a change in context light, tapping related activity increasing for successive hits and context attention increasing transiently, when context light changes). This means that an effect related to the belief of agency should be apparent in the main contrast OA-HA and in the same direction in the control contrast OA-HAC. An effect related to successful tapping performance should change gradually over time, the longer the rhythm was kept and thus should show opposite effects for the main contrast compared to the control contrast. Note that the context cue changed color at the start of OA and HAC, but not HA.

To test the hypothesis of multiple networks and functional mechanisms supporting the sense of agency and to investigate their dynamic contributions at the time of action-outcome, we used magneto-encephalography (MEG), which provides high temporal and good spatial precision. We investigated the changes in oscillatory activity and network activity during changes in the belief of agency in a continuous visuo-motor tapping task, as illustrated in Fig. 1. By inducing different causal beliefs in participants across states of matched sensorimotor contingencies, we could identify areas that responded specifically to this higher order belief without confounding factors like mismatch, attention or prediction error. Furthermore, by introducing a control state at the end of each tapping sequence, we could dissociate processes involved in tapping performance from processes related to the causal belief that should show the same modulation in the main and control contrasts.

Our data show that right EBA, right IPL, left primary motor cortex (lM1) and right MTG are keyplayers in this process, changing their relative contribution as reflected in distinct spectral profiles and connectivity patterns. First, during the belief of agency, EBA and MTG show reduced local feed-forward processing as indicated by specific gamma band suppression. Second, lM1 increases functional connectivity with rIPL and rMTG, indicated by increased beta band coherence, during self-attribution of an external event. Third, changes in external agency cues activated another oscillatory network, connecting lM1, insular and right SMA/vPMC in the alpha band, a circuit that supports active inference in social action contexts^5^. Finally, in parallel, but independent of agency related processes, cerebellum and EBA seem to support rhythmic tapping performance, with more suppression in the alpha and beta band during better rhythmicity.

## Methods

### Participants

Twenty-six healthy volunteers participated in this study (13 female, mean age 24 ± 4). Monetary compensation was provided for their participation (10 €/hr.). The ethics committee of the Medical Association Hamburg approved the study. Informed consent was obtained from all participants prior to each recording, and the experiment was carried out in accordance with the approved guidelines and regulations.

### Setup

Participants were seated in a chair positioned in a magnetically shielded and sound attenuated room. The experimental setup consisted of a set of 3 fiber optic lights (Omron e3x-na), which (i) presented a rhythmic white light stimulus straight ahead (flash duration <1 ms, 1.5 Hz), (ii) provided a continuous context or “state” light ~6 degrees to the left of the participant (green or red, counterbalanced across participants; see “Experimental paradigm” section), and (iii) served as a light barrier located on the right arm rest to record tapping performance of the index finger. A matlab-based customized program was used to control the stimuli and control the state transitions dependent on the participants’ current tapping performance.

### Experimental task

Participants were asked to fixate a rhythmically flashed light stimulus and tap synchronously with this stimulus such that the impact of their right index finger on the armrest coincides with the flash. Participants were instructed to keep the rhythm themselves in case of a change in the context light (6 degrees to the left), now causing instantly the flash with their tap (delay <1 ms). Participants started in the NO AGENCY (NA) state (Fig. 1) with the rhythmic light flashing (duration <1 ms; frequency 1.5 Hz) independently from their motor response and they had to synchronize their performance. After four successive hits (i.e. tap within 75 ms window of opportunity around time of the flash), participants would enter the next state, called HIDDEN AGENCY (HA), were the flash was already time-locked to the impact of the index finger as long as the participant still hits the window of opportunity (Fig. 1A), but without a change in the context light. This state was not explained to the participants and due to the small window of opportunity they should not become aware of this manipulation, which was validated by a questionnaire at the end of the experiment. Crucially, as soon as a participant did not tap precise enough (‘miss’), the state switched instantly back to NA to prevent him from noticing this hidden state. After four hits in the HA state, the context light would now indicate that they were actually agent over the flash (OVERT AGENCY, OA) (Fig. 1B). Importantly, this setup allowed us to obtain trials that have the same sensorimotor contingencies, as in both HA and OA the flash was locked to the motor response but the two states were associated with different causal beliefs. Finally, after six to nine hits in the OA state, the state of hidden agency was repeated (HIDDEN AGENCY CONTROL, HAC), with the context light changing color again to (misleadingly) indicate loss of agency for four hits and then the sequence starts over again. Note that a ‘miss’ in any state would instantly cause a switch back to NA, therefore, the later the state in the fixed sequence from NA to HA to OA to HAC, the more successive hits were needed to reach it, or in other words, the more difficult it was to reach (see Fig. 1C for an illustration of that argument).

### Training

Initially, this performance was trained (maximum 15 min.) and participants had to show that they were able to hit a 50 ms wide window of opportunity centered on the time of the flash (considered a ‘hit’) successively for 10 trials. During this training a feedback tone was provided that turned on as soon as they were performing hits. Participants who did not succeed were excluded from the experiment, as a rather sophisticated tapping performance is necessary to prevent participants from noticing the upcoming experimental manipulation. During the experiment, the window of opportunity for a tap to be considered a hit was 75 ms long and centered on 2/3 s after the last tap to allow slow drifts.

Debriefing and control questionnaire. Participants had to fill in a questionnaire at the end of the experiment, asking questions like “Did you notice anything unusual during the experiment?”, “Did you always experience agency when the context light indicated so?” or “Could you easily synchronize to the flash?”. Four participants were excluded because they occasionally suspected to have caused the flash, even when the context light did not change color. Some participants thought in the hidden agency state (HA) that we manipulated the rhythmicity of the flash to make it more difficult, but were unaware of causing the flash themselves. In total 26 dataset were used for the analysis. Participants performed 12 blocks of 180 trials (2 min. block duration), with self-paced breaks in between. In total 2160 trials were recorded per participant. The number of trials in each state depended on the performance of the participant.

### MEG data

MEG data were recorded continuously using a whole head system with 275 axial gradiometers (CTF275 Systems, VSM MedTech). Head position with respect to the sensor array was measured using localization coils fixed at anatomical landmarks (nasion, and left and right ear). MEG signals were sampled at 1200 Hz (low-pass filtered at 300 Hz) and then saved to disk. Line-noise was removed offline with notch filters (at 50, 100 and 150 Hz) and data was downsampled to 300 Hz after lowpass at 150 Hz.

### MRI data

T1-weighted high-resolution structural images (MRI) of each participant were acquired with a Siemens MAGNETOM Trio Scanner using a coronal magnetization-prepared rapid gradient echo sequence. These anatomical MRIs were recorded with anatomical reference markers at the same locations as the head position coils during the MEG recordings. These reference markers allow alignment of the MEG and MRI coordinate systems.

### Data analysis

We analyzed the data offline with open-source Fieldtrip software^34^. We applied semi-automatic artifact rejection of jumps, muscle artifacts (identified on highpassed data), drift from cars driving nearby and removed eye-movement and heartbeat related ICA components (identified from 64 components, by topography and spectral profile).

After artifact rejection for 254 (76), 437 (189), and 156 (110) trials were left over on average (SD) respectively, but trial numbers were stratified by random selection for the main and control contrast. Note that NA trials were not analyzed, because the sensorimotor contingencies differed. Tapping frequency, jitter and visuo-motor delays did not differ across HA, OA and HAC conditions (all p > 0.05), suggesting that attention did not differ between conditions. The average tapping frequency was 1.51, 1.53 and 1.52 Hz respectively, with standard deviations below 0.03 Hz in all analyzed states.

### Localization

To localize the neural sources of the spectral components of interest, an adaptive spatial filtering technique, Dynamic Imaging of Coherent Sources^35,36^ was used. Each participant’s brain volume was divided into an individually spaced three-dimensional grid using SPM8^37^ with each location corresponding to a location in the regular 1 cm grid based on a brain template (International Consortium for Brain Mapping; Montreal Neurological Institute (MNI), Montreal, QC, Canada). We warped every participant’s MRI to fit this template MRI and then inverse warped the template grid to obtain a grid in individual MNI coordinates for each participant. This procedure allowed us to directly compare grid points across participants in MNI space without the need to normalize. For each participant, the filter was computed from forward models with respect to dipolar sources at each individual grid point (the leadfield matrix) and the cross-spectral density between all combinations of sensors at the frequency of interest^38^. The computed spatial filter attenuates activity from all other locations and fully passes activity from the location of interest. We used single-sphere head models from each participant’s individual MRI to calculate the lead field matrix.

Time-frequency representations (TFRs) in source space were calculated based on a Fourier approach, applying a sliding window, with neighboring time points temporally segregated by 50 ms. To optimize time-frequency resolution, we analyzed separately two frequency ranges (2–30 and 30–90 Hz), as we did in our previous studies^39–41^. Based on well-known sensory attenuation and action-outcome binding effects of sense of agency, we decided to focus on data between the tap and the perceptual processing of the outcome (0–500 ms for low frequencies and 0–400 ms for high frequencies).

### Power calculation

First, we time-locked the data to each visuo-motor event (−1s to 1s) and calculated the combined planar gradient signal. After low passing the data at 35 Hz and trial-wise demeaning, we calculated the event-related field for each state. For our main analysis, induced power was calculated by subtracting the state-specific average event-related field from each trial separately. For the evoked power analysis, we analyzed the averaged data. We used a window of 500 ms post-tap and a Hanning taper for the lower frequencies, resulting in a spectral smoothing of ∼3 Hz. For the higher frequencies (30–90 Hz), we applied a multitaper approach^42^ using a window of 400 ms and 11 orthogonal Slepian tapers. This resulted in a spectral smoothing of ∼14 Hz. For time-resolved frequency analysis these windows were shifted in steps of 50 ms from −0.75 s to 0.7 s. Each window thus included action-outcome, but ended before the next tap. Note however, that action intention for the next trial is included, but state switch trials were analyzed separately. Each predefined time-frequency tile entered our subsequent statistical analysis at the source level.

Switch trial exploratory analysis was performed in the same way, with time-locked activity calculated in a longer window (−1 s to 2 s) and only included trials in which a switch trial had two trials of equal state before and after. Difference scores between the two switch-types were t-transformed per frequency-time tile across subjects to avoid baseline confounds.

### Statistical analysis of power

We used a nonparametric randomization procedure (montecarlo permutation) to control for multiple comparisons in voxel space (p < 0.05). As a first level statistic we computed t-values based on the difference of power values between states. These voxel-wise t-values cannot be interpreted as a statistical test outcome, but serve as inputs to the permutation test and the control analysis. The highest t-values were then compared against a permutation distribution (5000 permutations), with states randomly assigned, under the null hypothesis that assignments to states are exchangeable. Therefore, no further correction for multiple comparisons was necessary. Note that we did not use a cluster procedure here because significant clusters would form across distant local peaks, as spatial separation is never perfectly achieved by the beamformer and signal to noise was high for the power analysis. We excluded spatially isolated single voxel results though (4 in total), as they constitute most likely false positives.

Based on previous studies, we used predefined frequency bands, that have been shown relevant for sensorimotor processes and cognitive processes (alpha: 8–12 Hz and beta: 16–30 Hz) low gamma: 30–60 Hz; high gamma (60–90 Hz) and conscious perception^40,43–46^. We tested each frequency band separately for the main OA-HA contrast, identifying voxels that showed significant effects. Then for the control contrast OA-HAC, we constrained the post-hoc analysis to these voxels and applied a t-test over participants on the voxel-wise t-values to test if the modulation is in the same direction as the main effect or not (Bonferroni corrected for three frequency bands). We did this to reduce data dimensionality, because trial numbers were much smaller in that contrast, as it was the very last state participants could reach in the sequence.

For the alpha and beta band we also estimated trial-wise power values, pooled across states and correlated these with behavior (tapping frequency and number of previous hits at the current trial). We then applied a t-test across participants to test if these correlation values deviated significantly from zero (Bonferroni corrected for 5 regions of interest).

### Connectivity analysis

Connectivity analysis was constrained to the frequency bands that showed task related modulations in the previous analysis. Furthermore, here we report only on effects that do not show a power modulation in the same direction as the connectivity results, as this might confound the coherence analysis by a change in the reliability of phase estimates, even for imaginary coherence. The imaginary part of coherency removes the zero-phase lag interactions because these are entirely captured by the real part of coherency. This method is insensitive to leakage and excludes the contribution of instantaneous signal spread (artificial interaction). For further details see^38,47^.

We used time-locked data to obtain individual seed voxels of interest for the connectivity analysis. We time-locked the data to the visuo-motor events (see above) and reconstructed the data 0–55 ms (including early evoked responses in M1 and S1) post-tap in left primary motor cortex (lM1) voxels, based on automatic anatomic labeling, AAL^48^ by using the exact low resolution brain electromagnetic tomography, eLORETA software to compute the cortical three-dimensional distribution of current density in MNI source space^49^ after alignment of the MEG and MRI coordinate systems. The eLORETA method is a discrete, three-dimensional distributed, linear, weighted minimum norm inverse solution that has no localization bias, even with structured noise in the data. This was done for each voxel within the area separately. For each participant the voxel containing maximum power in the time-locked data was identified, under the assumption that early somatosensory evoked components, for example P50, evoked by the impact of the index finger, originate in the vicinity of the part of the motor cortex representing that finger. These coordinates were used as individual seed voxel coordinates for the connectivity analysis.

We calculated imaginary coherence for each participant, frequency band with task relevance (alpha, beta, low gamma) and state between the seed voxel (lM1) and all other voxels. We used a window of 500 ms post-tap and a Hanning taper, resulting in a spectral smoothing of ∼3 Hz, as for the power analysis. We calculated imaginary coherence in steps of 2 Hz for the alpha (8–12 Hz) and the beta band (16–30 Hz), averaging the obtained values across frequency steps. Then we calculated voxel-wise t-values based on the difference between states, which served as inputs to a cluster-based randomization procedure at the group level. In short, the spatial clustering procedure entails summing up spatially connected voxel-wise t-values that surpass a criterion (p < 0.05) and calculate the largest cluster-level statistic for all 5000 permutations and compare it to the largest cluster-statistic of the sorted data^50^.

As post-hoc control analysis (OA-HAC), we tested if there was a modulation of imaginary coherence within these voxels in the same or opposite direction, by means of a simple two-sided t-test across voxel-wise t-values. For this analysis, only participants with at least 150 trials in HAC were included (11 participants) to have a reliable estimate. Finally, we tested if power modulations within these voxels could explain these differences by applying a t-test on voxel-wise power-based t-values and if there was a power modulation in the same direction, we excluded the results.

## Results

### Power modulation

**Figure 2:**
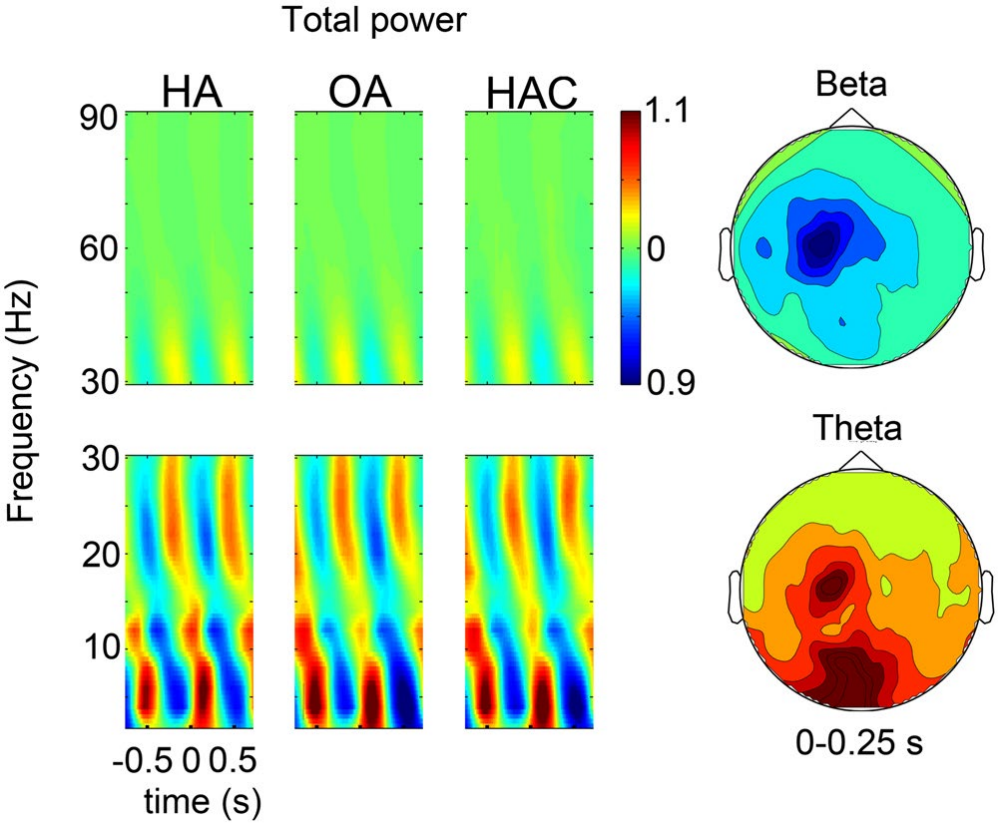
Total power at sensor level. The figure shows the time-frequency representation of normalized total power, during tapping, at ~1.5 Hz, time-locked to the current tap at 0 s. Left: Averaged power across sensors. The tap and time-locked flash at 0 s cause a transient decrease in beta band activity and an increase in the theta band. Right: The scalp distribution shows peak modulations across left motor cortex for beta band activity and over occipital sites for the theta band, averaged across conditions.

We first analysed total power at sensor level in the HA, OA and HAC trials. Figure 2 shows the time-frequency representation of total power, averaged across all sensors, with the current tap at 0 s. As expected for a rhythmic tapping task with visual stimuli, power modulations were strongest in the beta band and theta band over the left motor cortex and occipital sites. State specific evoked activity was subtracted from the data for further power and connectivity analysis, see supplementary information for details.

**Figure 3:**
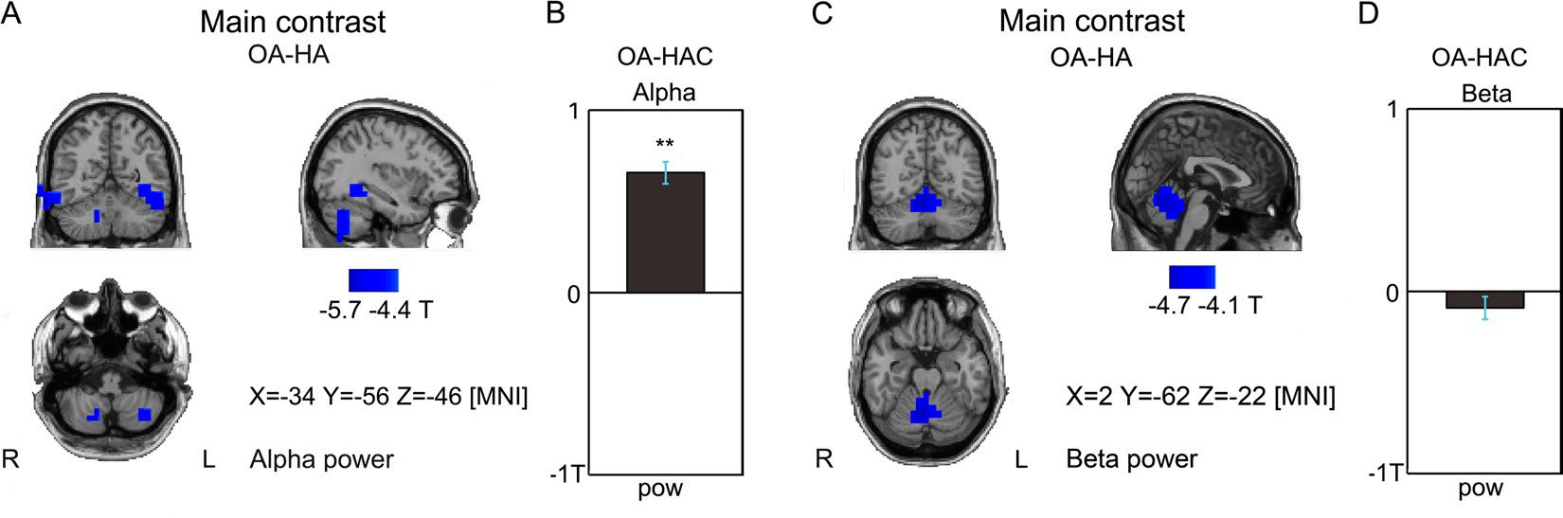
Induced alpha and beta power decrease during rhythmic tapping. (**A**) Alpha power (8–12 Hz) based t-values reveal significant differences in bilateral inferior temporal cortices and bilateral cerebrocerebellum, with less alpha power in OA than in HA (permutation test, p < 0.05). (**B**) Control contrast OA-HAC shows the opposite modulation in these voxels (T(101) = 10.8; p < 0.05; Bonferroni corrected). (**C**) Induced beta power decreases from HA to OA, but not from OA to HAC. Beta power (16–30 Hz) based t-values reveal significant differences in the spinocerebellum, with less beta power in OA than in HA (permutation test, p < 0.05). (**D**) Control contrast OA-HAC shows no significant modulation in these voxels (p > 0.05; Bonferroni corrected).

Subsequently, we analysed power changes in source space, focusing on differences between the HA and OA states. Figure 3A shows the induced alpha band modulation for the main contrast (OA-HA) in source space. There was a significant reduction of power in bilateral (inferior) temporal cortex (potentially including fusiform body area, FBA) and bilateral cerebrocerebellum (p < 0.05, permutation). The control contrast, however, showed the opposite modulation in these regions, with a significant increase, see Fig. 3B (T(101) = 10.8; p < 0.05, corrected). Thus, alpha power modulation was unrelated to the causal belief, but decreased over time as subjects pass sequentially through HA, OA and HAC during successful tapping (only hits). Furthermore, trial-wise correlation between alpha power and the number of previous successive hits deviated significantly from zero across participants for both regions (pooled across hemispheres), indicating more suppression for longer rhythmic tapping without misses (T = −3.8 and −3.2 respectively; p < 0.05 corrected).

We also observed significant condition differences for the beta band. Figure 3C shows a significant beta band suppression in the vermis of the cerebellum (and a small number of voxels in the caudate nucleus and the angular gyrus, not shown here) for the main contrast, OA-HA (p < 0.05, permutation), whereas there was no modulation for the control contrast OA-HAC, see Fig. 3D (p > 0.05). Thus, taken together the two contrasts do not indicate performance related effects as in the alpha band. Still, the trial-wise correlation between beta power and the number of previous successive hits deviated significantly from zero across participants in the cerebellum and caudate (T = −3.9 and −4 respectively; p < 0.05, corrected), but not angular gyrus (p > 0.05), indicating more suppression for longer rhythmic tapping.

**Figure 4:**
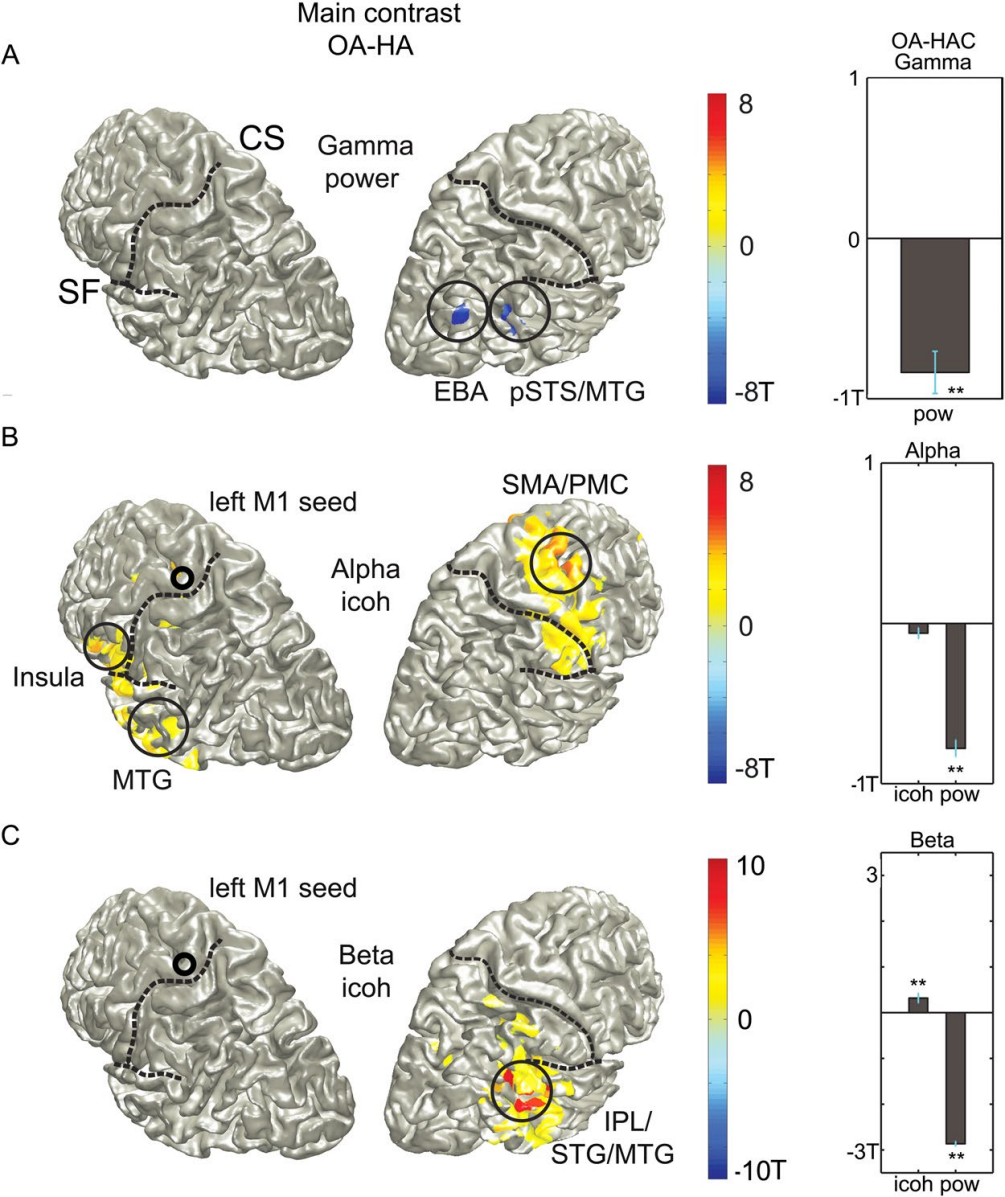
Induced gamma band power changes with belief of agency. (**A**) Left: Induced gamma band power (30–60 Hz) is significantly lower in OA than in HA at the occipito-temporal boundary, potentially extrastriate body area, and middle temporal gyrus (permutation test, p < 0.05). Right: Control contrast OA-HAC shows the same modulation in these voxels (T(24) = −6.4; p < 0.05; Bonferroni corrected). (**B**) Left: Left primary motor cortex connectivity differs significantly in the alpha band, with more connectivity for OA than HA to right premotor and left insular and temporal regions (permutation test, p < 0.05). Right: There was no difference for the control contrast OA-HAC in these regions (p > 0.05; Bonferroni corrected). Power modulations were in the opposite direction than imaginary coherence differences for the main contrast (T(1064) = −15.0; p < 0.05, corrected). (**C**) Left: Left primary motor cortex connectivity in the beta band (16–30 Hz) significantly differs between states, with higher imaginary coherence during OA than HA in parietal regions, partly overlapping with gamma band power modulations seen in (**A**) (permutation test, p < 0.05). Right: Post-hoc t-tests on voxels showing more connectivity for OA than for HA, show also significantly more connectivity for OA than for HAC (T(262) = 3.0; p < 0.05, corrected), which indicates a modulation by belief of agency. In contrast, there is significantly less power in the beta band for OA than for HA (T(262) = 54.8; p < 0.05) and for OA than for HAC (T(262) = 7.2; p < 0.05, corrected).

Furthermore, we observed gamma band power decreases with belief of agency in EBA and MTG. Gamma band power (30–60 Hz) shown in Fig. 4A was significantly lower in OA than in HA (p < 0.05, permutation) in the right middle temporal gyrus, extending into fusiform gyrus and in the caudal end of middle temporal gyrus adjacent to occipital cortex (MNI coordinates 52–62 8), most likely extrastriate body area (EBA)^33^. Importantly, the same modulation was observed for OA-HAC (T(24) = −6.36; p < 0.05, corrected), indicating a modulation by belief of agency and not performance. High gamma band power (60–90 Hz) showed no significant modulation (p > 0.05).

### Connectivity analysis

We analysed connectivity changes related to the main contrast between OA and HA by computing imaginary coherence in source space. Imaginary coherence between the seed voxel in left motor cortex and the rest of the brain differed significantly in the main contrast, as seen in Fig. 4B, with higher alpha band connectivity (cluster permutation, p < 0.05) between primary motor cortex and right pre-supplementary motor area (preSMA), premotor cortex (PMC), left anterior insula, left middle temporal gyrus, and both temporal poles (only visible from below) for OA than for HA. However, there was no significant modulation in either direction for the control contrast, as seen in Fig. 4B right column (p > 0.05), indicating neither a modulation by causal belief, nor performance. Within these voxels, there was a significant decrease in power for the main contrast (T(1064) = −15.0; p < 0.05).

In contrast, we observed significant beta band connectivity increases with belief of agency. Figure 4C shows a significantly higher beta band connectivity (cluster permutation, p < 0.05) between primary motor cortex and right inferior parietal, right middle temporal gyrus and right inferior temporal gyrus for OA than for HA trials. Importantly, the control contrast in the right column shows an effect in the same direction (T(262) = 3.0; p < 0.05, corrected), which indicates a modulation by belief of agency. Furthermore, power decreases significantly in these regions for the main contrast (T(262) = −54.8; p < 0.05, corrected) and the control contrast (T262) = −7.2; p < 0.05, corrected) and therefore the changes in connectivity are not confounded by better phase estimates due to power modulation. Interestingly, these connectivity changes partly overlapped with the gamma band decrease in middle temporal gyrus/inferior temporal gyrus.

### Time-course of the belief of agency

**Figure 5:**
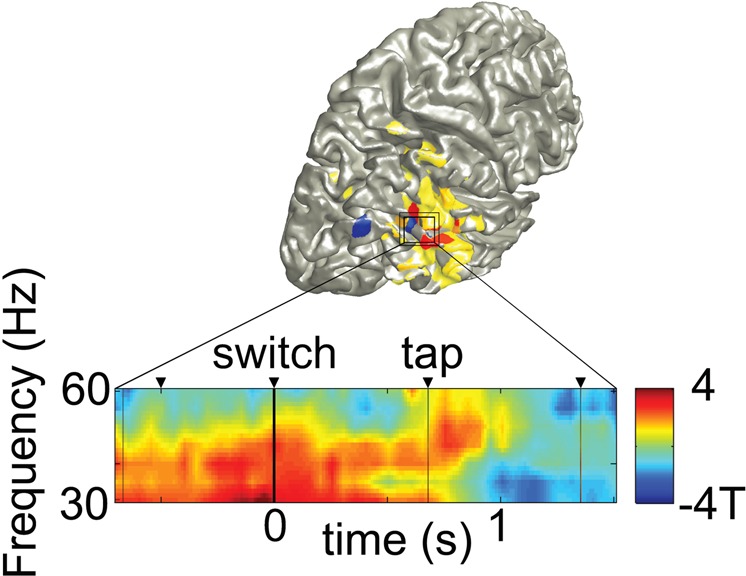
Beta connectivity (Fig. 4C) and gamma power (Fig. 4A) plotted on top of each other, with temporal evolution of the gamma band effect (MTG cluster of Fig. 4A) during state changes. The gamma band reduction (30–60 Hz) associated with the belief of agency is apparent only after the next tap and not at the current switch tap. Depicted are difference t-values across subjects for the two switch trial types (HA- > OA) - (OA- > HAC).

Figure 4A showed two regions in which gamma band power (30–60 Hz) was suppressed during belief of agency, one more posterior region, most probably EBA and the other in inferior/middle temporal gyrus, overlapping partly with beta connectivity increases during belief of agency in MTG, as shown in Fig. 5 with both clusters (Fig. 4A,C) plotted on top of each other. To investigate the time-course of this agency related effect in the latter region, we calculated time-varying power modulations in these voxels, now contrasting switch trials in which belief of agency is obtained after the switch, with those where it is lost (HA/OA)-(OA/HAC). As can be seen in Fig. 5, the modulation of gamma band power is only apparent after the next tap (at 0.66 s), in contrast to a direct modulation caused by the change in context light at 0 s.

## Discussion

We studied the belief of agency from a network perspective, investigating changes in local oscillatory activity in MEG power spectra as well as connectivity changes as assessed by imaginary coherence between motor cortex and the rest of the brain. We manipulated the belief of agency for conditions with the same sensorimotor contingencies and were able to dissociate performance (i.e. tapping) related effects from effects related to the belief of agency.

We found a significant gamma power decrease in right MTG and EBA related to the belief of agency. Recently, a well-controlled fMRI study found several regions to be sensitive to sense of agency during visual feedback manipulations of hand movements, with the highest modulations in right MTG, STG and MFG^28^. Interestingly, they found BOLD decreasing proportionally with the sense of agency in these regions, which is in line with the gamma decreases observed with the belief of agency in our study, given the reported positive correlation between gamma band activity and BOLD^51^. Furthermore, our effects did not only include EBA and MTG, but also extended into inferior temporal lobe, potentially fusiform body area (FBA), and pSTS, in line with previously reported multimodal body representations^21^.

On a neurophysiological level, gamma band activity has been related to feed-forward processing in the visual hierarchy and increased gain modulation^35,36^. Therefore, our observations might reflect more local feed-forward processing of bodily representations when participants aim to keep their behavior synchronized to an external event, without the belief of causing that event. A recent TMS study suggests that EBA calculates future postural states and its computations have been shown particularly important during discrepancies between desires and current state^52^. Moreover, gamma band activity has been shown to reflect processing of prediction errors in sensorimotor cortex and might be involved in somatosensory perceptual attenuation induced by action^53^. Furthermore, EBA activity showed agency-error specific activation in a paradigm that controlled for general prediction errors^18^ and is modulated by the self-other distinction^18^. In our paradigm, however, there was no error or mismatch, as the intended action-outcome (synchronous tapping with the flash) was achieved perfectly in both conditions, given our hidden experimental manipulation. Both states have perceptual events time-locked to the motor response. Also tapping behavior did not show any differences in frequency or jitter.

Taken together, this leads to the interesting hypothesis that the causal belief or the intentional state changed the mismatch detection process in these regions. In other words, the comparison process is reduced during the belief of agency, as reflected in suppressed gamma band activity in areas computing future postural states and their visual predictions. These observations might also explain various findings showing perceptual distortions during self-attribution of a perceptual event^30–32,54,55^.

Interestingly, further investigation of the time-course of this effect in our data suggests that the brain state changes only after the next event is processed, and not with the context cue change. This is in line with the role of IPL in agency estimation at the time-point of action selection, shown recently with TMS^23^ and the neural binding of action and outcome by intention as the basis for sense of agency^6^.

In sum, agency dependent processes have been difficult to disentangle from more general mismatch detection networks, because sense of agency was manipulated by mismatches of action and outcome. In contrast, our results show that the causal belief changes activity in structures involved in the predictive comparison process.

Importantly, we also observed connectivity changes related to the belief of agency, showing more communication of motor cortex with MTG and IPL. While local gamma band activity in MTG and EBA decreased, motor cortex increased its connectivity in the beta band with right MTG during the belief of agency, but not EBA. Beta band coherence has been shown to be involved in feedback processing in the visual hierarchy in macaques^56^ and humans^57^. In general, it is often involved during distant communication in the brain^43,58^.

Thus, our results suggest that the feedforward activity of rMTG, reflected in local gamma band activity, is replaced by long-distance beta band connectivity reflecting increased recurrent processing with motor cortex. Interestingly, not only MTG increases its communication with motor cortex during that state, but also inferior parietal cortex. This is in line with previous work on the sense of agency, which has suggested an involvement of IPL in a large variety of agency paradigms^6^. Moreover, a TMS study on inferior parietal cortex has provided causal evidence for the role of IPL in the sense of agency. Phase synchronization patterns^27,59^ and local oscillatory activity patterns^60^ were interpreted as the involvement of IPL in the sense of agency and visuotactile congruency in the rubber hand illusion increases parietal interelectrode synchronization in the gamma band^61,62^, but data analysis was not performed at source level in these studies. A recent DCM study also suggested increased coherence between parietal and frontal nodes during sense of agency^63^, but sensorimotor contingencies (deviation angles) were not matched in that study.

Taken together, our data suggest that the belief of agency reduces EBA involvement and increases recurrent processing between motor cortex, MTG and IPL, which might reflect reduced agency-specific predictive comparison processes during that state.

Alpha band connectivity between motor cortex, insula and (pre) SMA/PMC increased only for the main contrast, between motor cortex, insula, temporal poles and SMA, but not for the control contrast. Therefore, this effect cannot be attributed to a given causal belief, nor to performance in our data, even though there are studies reporting a contribution of alpha band activity to the sense of agency^45,46^. One possible interpretation of that finding is that the change in context cue at the start of OA and HAC, but not HA (Fig. 2B), drove this effect. We excluded switch trials for these main analyses, because we were interested in a steady cognitive state, with the subject either having a belief of agency or not. If the change in action context, however, caused transient neural effects lasting longer than one tap-cycle, the difference observed only in the main contrast OA-HA might be induced by a context change at the start of one but not the other condition.

In line with this interpretation, (pre-) SMA/PMC and medial frontal cortex are part of the network proposed to instantiate active inference during motor tasks^5,18^ and a recent dynamic causal modelling study suggested pre-SMA modulation by inferior parietal cortex during sense of agency^63^. Furthermore, medial frontal gyrus and SMA activity have been shown to respond to judgment of agency^18,28,55,64,65^. Dorsolateral prefrontal cortex, however, did not appear in this contrast. One reason might be, that action selection is not involved in our agency task^66^. Another reason might be the higher prefrontal anatomic variability across subjects and increased distance to the sensors in comparison to other sites. Insula has been shown to reflect social-action context evaluation, providing a convergence point between external/internal milieus^4,19,28,65,67,68^, whereas temporal poles might mediate target action context processing^5^. Therefore, this network might support active inference during agency estimation when there is a change in an external agency cue.

A better performance in this task (staying longer in the rhythm) was related to alpha power modulations in the bilateral cerebellum and potentially fusiform body-areas, with less power for a more successful performance. Alpha band activity has been shown to index excitability in various structures of the visual hierarchy (excitability hypothesis)^46,69,70^, as well as in sensorimotor networks^41^. Beta band activity in contrast, has been strongly related to excitability of the motor hierarchy^71^, but also the dorsal attention network^72^ and tactile attention^73^.

Interestingly, the alpha band modulation in inferior temporal cortices and the cerebrocerebellum was observed bilateral and both structures contain body representations and cerebellum has been shown to predict sensory consequences of actions^74^, which might be particularly relevant in our task. Whereas alpha band modulations in bilateral fusiform regions and cerebellum have not been reported earlier to our knowledge, the gradual decrease throughout a successful tapping sequence suggests that the alpha band excitability hypothesis might hold in these structures as well.

Whereas the contribution of the cerebellum to sensory processing is a rather new finding, this structure is well known for its involvement in motor planning, timing and coordination^75^. In contrast to the bilateral cerebrocerebellar modulation, beta band activity was modulated in the spinocerebellum, a structure that supports rhythmic and ongoing movements. Even though we used a tapping frequency that is considered a discrete mode (<2 Hz) in the literature^76,77^, to obtain discrete efference copies and agency related processes, the high temporal precision required in this task might enforce more ongoing movement characteristics. Clinical research in patients with Parkinson’s disease has revealed that beta band activity plays a major role in rhythmic tapping behavior^78^ and reduced beta band power has been associated with better performance^79^, which fits nicely to our observations.

To summarize, we have shown that studying belief of agency with MEG provides us with unique insights onto the formation of transient networks between structures well known for their involvement in the sense of agency. Our results highlight two distinct processes that are characteristic for the belief of agency. First, primary motor cortex shows stronger coupling to inferior parietal lobe and right middle temporal gyrus via recurrent long-distance communication mechanisms (beta band connectivity) and second, the local feed-forward activity (gamma band power) in EBA and middle temporal gyrus disappears in this state, a change that takes place after the next action. These findings might provide the neural correlates of reduced predictive comparison processes when an event is attributed to oneself, potentially provocing previously observed action-outcome binding effects and perceptual distortions. The stronger communication between motor cortex, IPL and temporal regions, but not EBA might reflect their increased contribution in that state, supporting the role of the active agent. Additionally, overt changes in social action context seem to increase feedback mechanisms (alpha band connectivity) between primary motor cortex, medial frontal, premotor and left insular, as well as temporal regions, a network that might support active inference in changing social action contexts.

We conclude that the neural basis of the sense of agency is best characterized by a complex network account with multiple hubs involved at various cognitive levels, from sensorimotor predictions to cue evaluation. Our data suggest that the causal belief changes the relative contribution of the keyplayers involved in the process across the hierarchy, as seen in their spectral fingerprint and communication patterns. These findings might explain a wide variety of behavioral findings and contribute to a more elaborate view on the complexity of brain networks supporting the sense of agency.
